# A Two-Year Treatment of Amnestic Mild Cognitive Impairment using a Compound Chinese Medicine: A Placebo Controlled Randomized Trail

**DOI:** 10.1038/srep28982

**Published:** 2016-07-04

**Authors:** Junying Zhang, Zhen Liu, Huamin Zhang, Caishui Yang, He Li, Xin Li, Kewei Chen, Zhanjun Zhang

**Affiliations:** 1 State Key Laboratory of Cognitive Neuroscience and Learning & IDG/McGovern Institute for Brain Research, Beijing Normal University, Beijing 100875, P. R. China; 2 BABRI Centre, Beijing Normal University, Beijing 100875, P. R. China; 3 Institute of Information on Traditional Chinese Medicine, China Academy of Chinese Medical Sciences, Beijing 100700, P. R. China; 4 Banner Alzheimer’s Institute, Phoenix, AZ 85006, USA

## Abstract

We aimed to investigate the long-term therapeutic effects of a compound Chinese medicine, the Bushen capsule, on cognition and brain connectivity in patients with amnestic mild cognitive impairment (aMCI). Thus, sixty aMCI participants were recruited to this 24-month study and were randomly divided into treatment (30 with a Bushen capsule) and placebo (30 with a placebo capsule) groups. Neuropsychological tests with MMSE and episodic memory as the primary outcomes and resting-state functional magnetic resonance imaging (fMRI) were analyzed before and after the treatment over 24 month period. In contrast to the placebo group, the drug group presented improved or stable general cognitive function, memory, language and executive function especially the primary outcomes MMSE and episodic memory with Bushen capsule treatment. FMRI results showed increased connectivity in the right precuneus and the global connectivity indexed with goodness of fit (GOF) of the default mode network (DMN) in the drug group and decreased GOF in the placebo group. More importantly, we found the GOF change was positively correlated with changes in MMSE and memory scores after 24 months in the drug group. Over 24 months, treatment with the compound Chinese medicine Bushen capsule can improve multiple domains of cognition and increase the functional local (right precuneus) and global connectivity within the DMN, which are associated with better performance.

Mild cognitive impairment (MCI) is considered to be an intermediate phase between normal aging and dementia^1^. Slowing down or even reversing the process of MCI could aid the early intervention and the ultimate prevention of Alzheimer’s disease (AD). Tremendous efforts have been made worldwide to develop and evaluate potential treatments for AD^2^,^3^,^4^,^5^,^6^. Despite many challenges and setbacks, efforts to cure AD including MCI continue.

Among the investigated treatments are those based on traditional Chinese medicine (TCM), which has sought after for its potential in the prevention and cure of amnesia because of its uniqueness and holistic view of treating diseases^7^. One theory in the TCM system, the “BuShenYiZhi, JieDuTongLuo” theory, plays an important and principal role through regulating tissue microenvironment and recovering neurological function. Bushen capsules (BSCs) are classic TCM formulas that are composed according to the “BuShenYiZhi, JieDuTongLuo” theory. Evidences have supported that the BSCs could relieve the accelerated cognitive impairments such as memory decline in MCI and dementia^8^,^9^. The main components, including *Cistanches Herba, Rhizoma Alismatis*, and *Polygonum multiflorum thumb*, have been reported for their effects on improving learning and memory^9^,^10^,^11^,^12^,^13^. This study, built on the findings of its short term effects^14^,^15^, is intended to establish the long-term effects of BSCs on cognitions.

In addition, functional magnetic resonance imaging (fMRI) has been widely used in the studies of AD and AD risks^16^. The spontaneous fluctuation signals in the resting state brain delineate the human neural functional architecture as multi-networks such as the default mode network (DMN), frontal–parietal network, somatomotor network, and primary visual network^17^. Among these networks, DMN is the most reported networks in the AD literatures^18^,^19^. In fact, studies have shown that the connectivity within DMN changed in AD and MCI patients^18^,^20^. Thus in the present pilot study, we used a double-blind placebo-controlled design and resting state fMRI to study the long-term effects of TCM on cognitive functions with MMSE and episodic memory as our primary outcomes (due to the reported main effects of the Bushen capsules’ components on improving memory^9^,^10^,^11^,^12^,^13^) and connectivity patterns of DMN in amnestic MCI (aMCI) patients undergoing a baseline, following 12 and 24 month visits. The purpose of this study was to evaluate: (a) whether the drug could maintain or improve behavioral performances, in general cognitive function especially MMSE and episodic memory; (b) and whether the drug could improve intrinsic functional connectivity in DMN after treatment.

## Results

### Interactive effect of group × time in the neuropsychological results

At baseline, no significant differences were observed in age, gender, years of education and *APOE* ε4 allele frequency between the drug group and the placebo group (*P* > 0.05) (Table 1).

**Figure 1:**
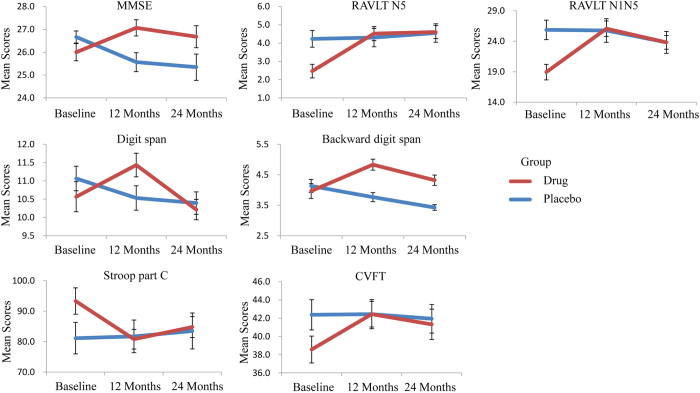
Line graphs showed significant interactions of group × time in the neuropsychological assessments. Error bars denote standard error of the mean.

To evaluate the drug efficacy, 2×3 ANCOVA for the neuropsychological tests were conducted. For the primary outcome measures, the general cognitive function (MMSE, *P* < 0.001), and episodic memory (RAVLT N5, *P* = 0.001 and RAVLT N1N5, *P* = 0.001) showed significant interactions between group and time after controlling for age, gender, and years of education (Table 2, Fig. 1). Additionally, the general cognitive function (MMSE, *P* = 0.046) showed significant group effects (Table 2). The effect of interactions showed that the general cognitive function (MMSE, *P* = 0.019), and episodic memory (RAVLT N5, *P* < 0.001 and RAVLT N1N5, *P* < 0.001) changed significantly in the drug group along with the treatment time. The mean values of the above tests after 12 months and 24 months were increased or stable compared to the baseline measures. Meanwhile, the general cognitive function (MMSE, *P* = 0.002) changed significantly in the placebo group along with the treatment time. The mean values of both tests after 12 months and 24 months were decreased from the values at baseline. At baseline, the episodic memory (RAVLT N5, *P* = 0.003 and RAVLT N1N5, *P* = 0.002) scores in the drug group were lower than in the placebo group due to the random allocation of the subjects. After 12 months of treatment, the general cognitive function (MMSE, *P* = 0.015) scores in the drug group were higher than scores in the placebo group.

For the secondary outcome measures, the working memory (Digit span, *P* = 0.018 and Backward digit span, *P* < 0.001), language function (CVFT, *P* = 0.011) and executive function (Stroop part C, *P* = 0.048) showed significant interactions between group and time after controlling for age, gender, and years of education (Table 2, Fig. 1). Additionally, the working memory tasks (Backward digit span, *P* = 0.025) showed significant group effects, and the language function (CVFT, *P* = 0.041) and processing speed (TMA part A, *P* = 0.004) showed significant time effects (Table 2). The simple effect of interactions showed that the working memory (Digit span, *P* = 0.003 and Backward digit span, *P* < 0.001), language function (CVFT, *P* = 0.030) and executive function (Stroop part C, *P* = 0.004) changed significantly in the drug group along with the treatment time. The mean values of the above tests after 12 months and 24 months were increased or stable compared to the baseline measures. Meanwhile, the working memory (Backward digit span, *P* = 0.001) changed significantly in the placebo group along with the treatment time. The mean values of both tests after 12 months and 24 months were decreased from the values at baseline. After 12 (Backward digit span, *P* < 0.001) and 24 (Backward digit span, *P* < 0.001) months of treatment, the working memory scores in the drug group were higher than scores in the placebo group. None of the other contrasts showed any significant differences (all *P* > 0.05).

### Interactive effect of group × time in DMN

#### The functional connectivity within DMN

**Figure 2:**
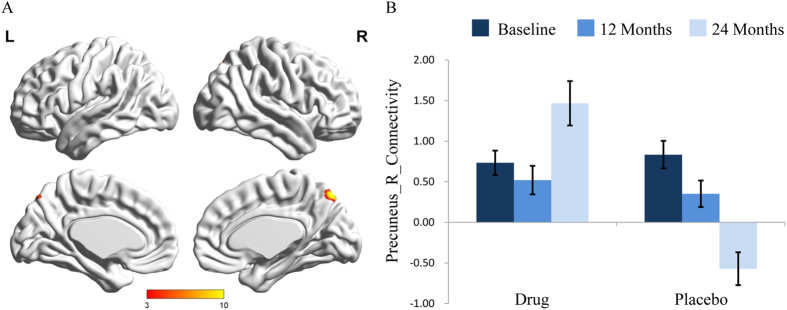
(**A**) Interactive effect of group × time in the functional connectivity within the DMN showed the area in the right precuneus (x = 9 mm, y = −75 mm, z = 51 mm; voxel size = 55). The x, y, z coordinates of the primary peak in MNI space. (**B**) The bar graph shows the ROI analysis on the significant regions from voxel-wise comparisons. Error bars denote standard error of the mean. DMN, default mode network; ROI, region of interest.

For the nueroimaging perspective, the DMN were identified from the results of the group ICA; Supplementary Figure e-1 describes the anatomical regions involved for both groups at all three time points. A full factorial ANCOVA (2 × 3) was conducted with time as the within-subject factor and group as the between-subject factor to identify changes within the DMN after for controlling age, gender, and years of education. This analysis revealed a significant interaction effect of group × time on the brain region connectivity of the right precuneus (x = 9, y = −75, z = 51, AlphaSim-corrected, *p* < 0.001, Fig. 2A).

The simple effects of group and time were further analyzed (Fig. 2B). After 24 months of treatment, the functional connectivity in the right precuneus increased significantly in the drug group (F = 3.75, *P* = 0.032), whereas functional connectivity decreased significantly in the placebo group (F = 11.80, *P* < 0.001). For the baseline and 12 month visit, the functional connectivity in the right precuneus did not show differences between both groups (F = 0.45, *P* = 0.511 at baseline, and F = 0.70, *P* = 0.486 after 12 months). This effect was significant for the 24 month time period where the connectivity in the right precuneus was increased in the drug group compared to the placebo group (F = 16.56, *P* = 0.001).

#### The GOF index

**Figure 3:**
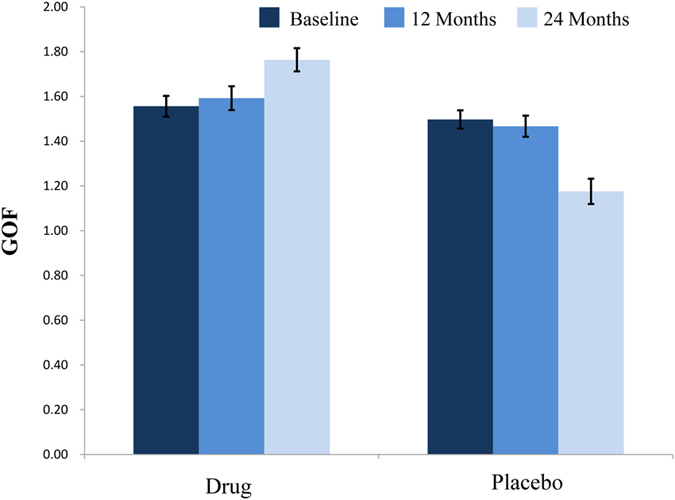
The bar graph shows the interactive analysis of group × time in the GOF index. Error bars denote standard error of the mean. GOF, goodness-of-fit.

In addition to the voxel-wise analysis that identified interactive effects at the right precuneus, we utilized the global index GOF (goodness of fit). The results showed a significant interaction effect of group × time (F = 15.683, *P* < 0.001) (Fig. 3). The simple effect of time showed that the GOF index increased in the drug group (F = 5.16, *P* = 0.010), whereas it decreased in the placebo group (F = 13.61, *P* < 0.001). The simple effect of group showed that the GOF index did not show differences between both groups at the baseline and after 12 months (F = 0.66, *P* = 0.425 at baseline, and F = 0.87, *P* = 0.362 after 12 months), but showed differences after 24 months (F = 39.60, *P* < 0.001).

### Correlations between GOF index and cognitive function

**Figure 4:**
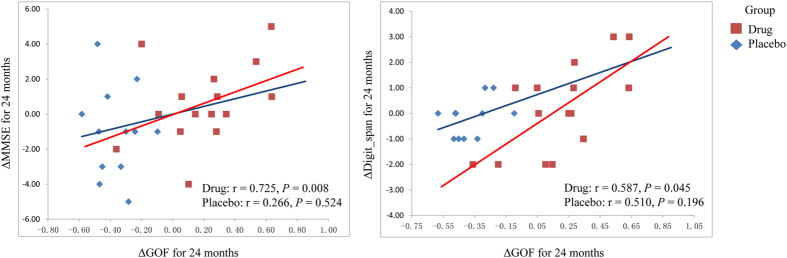
Changed cognitive functions were associated with changed GOF index in the DMN. GOF, goodness-of-fit; DMN, default mode network.

To assess whether the functional network differences could explain the changed cognitive functions, we correlated the changed connectivity measurements of the significant clusters from the voxel-wise comparisons within the DMN and the GOF index with cognitive performances of significant interactive effects. In the drug group, pearson correlation analyses indicated significant positive correlations between changed GOF index and changed MMSE scores (r = 0.725, *P* = 0.008), and changed digit span scores (r = 0.587, *P* = 0.045) for 24 months (Fig. 4). No significant correlations were found in the placebo group for changed values of 24 months. Correlation analyses between changed network indexes and changed cognitive performances for 12 months showed no significant effects. However, there were no correlation results survived *p* < 0.05 after FDR correction for multiple comparisons.

### Incidence of adverse events

Incidence of adverse events was the same in both groups, with no significant difference (*P* > 0.05) in the incidence of adverse events. During the first three months of the medication, one participant from treatment group had a decreased appetite after taking medicines for 3 days. Moreover, one participant from the placebo group had mild nausea after taking the placebo for one week. Neither of them received special treatment and these symptoms faded away 4 days later; both participants continued to take the medicine. During 24 months after the intervention, a higher number of participants taking BSCs than those taking placebo experienced constipation, 7 (23%) vs. 4 (13%). No participants in the drug and placebo groups discontinued treatment because of an adverse event. The results of laboratory tests and physical examinations showed no clinically significant differences among groups between baseline and the final visit. No participants, neither in the drug group nor in the placebo group, converted to AD.

## Discussion

Findings from our current study showed that the treatment arm, in contrast to the placebo arm, exhibited improved or stable cognitive performances in several cognitive domains, including our primary outcomes in general cognitive function, and memory as well as executive function and language ability. No MCI patients converted to AD during our 24-month trial period, consistent with results reported in a previous study^21^. The main reasons may be that those participants were recruited from the local community and at the disease’s early stage. In our subsequent analysis of DMN properties, we found that the regional connectivity in the right precuneus and the global GOF index increased gradually with prolonged treatment in the drug group, but declined in the placebo group. More importantly, the changed GOF index were correlated with changed cognitive performances over 24 months.

The primary findings in the current study were that the aMCI patients exhibited improved cognitive functions after BSCs treatment, manifested particularly for our primary outcomes in general cognitive function, and episodic memory. Besides, the working memory, executive function, and language ability got improved after treatment. As a test for general intellectual ability, MMSE is usually used for assessing basic cognitive status of patients, with high efficiency and sensitivity^22^,^23^. In a reported long-term trail of the donepezil, MMSE was also used as a measure to assess cognition^24^. Additionally, RAVLT has long been used as an ideal evaluation index for episodic memory^25^. It is interesting, as shown in the Results section, to note that though the episodic memory of the drug group was worse than the placebo group at baseline, before the start of the study, the drug group exhibited comparative performance after 24 months treatment to that of the placebo group, indicative of a positive effect of the drug on aMCI patients’ episodic memory. We found similar results from mild AD patients after 12 months of brain reperfusion rehabilitation therapy^26^. Evidences from previous studies showed that working memory (measured by Digit span and Backward digit span)^27^, executive function (measured by Stroop color word interference)^28^, and language ability (measured by CVFT)^29^ were associated with better performance and/or slower decline after drug treatment (including hormone and etanercept), which were consistent with our findings. Thus we would suggest that the BSCs could ameliorate the cognitive declines of aMCI patients.

Studies using an fMRI technique to investigate the therapeutic effect of TCM have been reported in recent years^14^,^15^,^30^,^31^,^32^,^33^. Among these studies, our previous pilot works demonstrated that MCI patients in the drug group showed ameliorative brain activation during a working memory task^15^ and an episodic memory task^14^ after 3 months of treatment, indicating improving positive effect of BSCs on working memory and episodic memory. Moreover, the precuneus has long been regarded as a core region of the DMN^34^. Compared to healthy elderly individuals, a previous study showed that MCI patients exhibited decreased functional connectivity in the precuneus within the DMN^35^. A longitudinal study showed that the functional connectivity in the precuneus was reduced in MCI patients compared to matched controls after a 20 month follow up^36^. Findings from all these earlier studies were consistent with our current results that the precuneus plays a crucial role in the process of AD and could become a sensitive brain region for clinical treatment. In our results, we found that the functional connectivity in the precuneus increased significantly in the drug group but decreased in the placebo group after two years of treatment, which could be suggestive that the BSCs therapy had a curative effect on cerebral functions.

The GOF, a quantitative global index of the DMN connectivity, has been shown to distinguish AD from healthy aging^37^. In addition, Petrella *et al*. found that the GOF indices were lowest in AD, intermediate in MCI, and highest in normal elderly individuals^38^. Consistently, we found that the GOF index increased in the drug group but decreased in the placebo group during the follow-up period. Thus one could speculate that the BSCs therapy improved the connectivity and efficiency of resting state DMN.

In the subsequent correlation analyses, we found that the changed DMN indices were positively related to changes in behavioral performance, including general cognitive function, and working memory in the drug group. The results suggested that accompanied by the ameliorative global network efficiency in the DMN, the behavioral performance improved significantly after two-year treatment. The two cognitive domains were consistent with our previous study, which indicated that the ameliorative brain functions were related to improve behavioral performance in aMCI patients after a 3-month treatment^15^. Additionally, the DMN is responsible for the performance of most cognitive functions, such as MMSE^39^, and working memory^40^. Although the correlation results did not survive FDR correction (*p* < 0.05), we could find a trend that better network efficiency indicated ameliorative cognitive functions.

The exploitation of Chinese medicine based on “BuShenYiZhi, JieDuTongLuo” theory has shown positive therapeutic effects on a broad spectrum of diseases including cancer^41^ and cerebral ischemia reperfusion injury^42^. The BSCs was developed according to this theory and included some components that could improve learning and memory, such as *Cistanches Herba, Rhizoma Alismatis*, and *Polygonum multiflorum thumb*. Through inducing nerve growth, improving effects of anti-oxidation and strengthening the immune system, *Cistanches Herba* and its extracts could improve learning and memory of dementia patients and memory-deficient rats^9^,^43^. *Rhizoma Alismatis* has been shown to ameliorate memory dysfunction, and protect the ultrastructure of the cerebral cortex due to aging and therefore has been used as a therapeutic TCM for senile dementia, especially AD^12^. Research on *Polygonum multiflorum thumb* and its extracts suggested it may have beneficial effects on hippocampal neurons through the suppression of reactive oxygen species accumulation^10^. As a compound medicine of Chinese herbs, studies have shown that combination of the component ingredients with proper proportions would provide better therapeutic effect than each component ingredient alone^44^.

Our study demonstrated that TCM could be promising multi-target drugs for AD treatment. A recent review details the roles of many bioactive components isolated from TCM on relieving disease symptoms, and suppressing amyloid-β (Aβ)-induced neuronal cytotoxicity and inflammation^45^. A recent study showed that the Chinese herbal formula, Yishen Huazhuo decoction, was superior to donepezil hydrochloride at improving cognitive performances in mild AD patients during a 24 week trial^46^, suggesting the strengths of TCM. One could speculate that “BuShenYiZhi, JieDuTongLuo” therapy could therefore be a promising therapeutic method for the treatment of AD, especially at early stages. This study suggested a further exploration of the significance of TCM in the MCI field.

This study had several limitations. First, the average age of MCI patients in the present study, who were around 65 years old, was younger than the age of those patients in usual clinical trials, which were usually in their early 70’s. Since cognitive abilities of brain vary with ages, our further studies would be done with the age variation of these participants and continue to monitor the drug efficacy on our participants. On the other hand, our study is inline with the increased focus on interventions at earlier phase of the disease including younger ages. Second, the ability of episodic memory (measures by RAVLT N5 and RAVLT N1N5) in the drug group was worse than the placebo group at baseline. Even though this cognitive function increased after treatment (i.e. we found no group differences in those two measures after 12 and 24 months), the differences at baseline could impact the final results. Thus, there are needs to verify the current results in more balanced samples. Third, as a multi-target drug, the specific active ingredients in the BSCs need to be figured out. Therefore, the efforts of pharmacologists should be studied in further work.

In summary, our current study suggested long-term ameliorative effects of the compound Chinese medicine BSCs on cognitive performances and DMN connectivity. To the best of our knowledge, this study is the first investigating the therapeutic treatment effects of this compound Chinese medicine based on “BuShenYiZhi, JieDuTongLuo” over a two-year period. Our findings showcase its efficacy in treating aMCI patients and demonstrate the feasibility of using neuroimaging biomarkers in clinical trials. Further studies with a larger sample size are needed to confirm our results and assess the values of these neuroimaging biomarkers.

## Methods

### Study design and patients

This clinical trial, with a double-blind placebo-controlled design, was registered in the Chinese Clinical Trial Registry, that was participated in the WHO International Clinical Trial Registry Platform, prior to its start (registration number: ChiCTR-TRC-12003073, registration date: November 5, 2012), and the study content was approved by the Ethics Committee of Dongfang Hospital, affiliated with Beijing University of Chinese Medicine. Over a 24-month period, the treatment group received medication at a frequency of 3 times a day, 4 capsules a time, while the placebo group received the same number of placebo capsules. Both the patients and investigators were blinded to the treatment allocation in the treatment and placebo groups until study completion. Throughout the medication course, vital signs and adverse event were recorded at the baseline, 12 and 24 month visits. In addition, laboratory tests and physical examinations were conducted at every visit. In addition, the methods we used were carried out in accordance with the approved guidelines.

**Figure 5:**
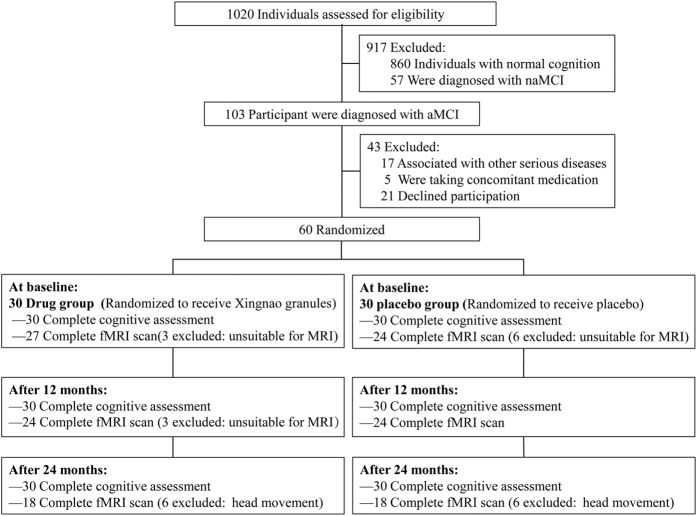
Participant flow chart.

The participants in the present study were from the Beijing Aging Brain Rejuvenation Initiative (BABRI) program and were recruited for a study of urban elderly adults without dementia in Beijing, China^47^. Of the 1020 patients screened, 103 were diagnosed with amnestic MCI (aMCI), 60 of them were included (Fig. 5) in the study based on the inclusion/exclusion criteria described below. All these 60 participants received neuropsychological assessments and a fMRI scanning by a professional imaging staff at the baseline, second visit (after 12 months) and third visit (after 24 months). The drugs used in this study were supplied by the sponsor. The appearance, smell and taste of the placebo capsules were disguised to be identical to the treatment drug. To ensure the quality of the cognitive testing, investigators were trained before the start of the study. The informed consent was obtained from all subjects. The demographic details of all the sixty aMCI participants (drug group, 30; placebo group, 30; average age 64.67 ± 6.83 years) were shown in Table 1. The trial inclusion criteria were as follows: 1. age from 50–80; 2. education of more than six years; 3. consisted with the Petersen’s criteria for aMCI which included^1^: subjective memory complaints; objective evidence of cognitive impairment and/or an objective examination that confirmed cognitive decline (in our study, we regarded subjects that demonstrated a deficit of Auditory Verbal Learning Test (AVLT) at least 1.5 standard deviation below the age-and education-adjusted as memory impairment or decline)^48^; normal activities of daily living; and not demented; 4. scores on the Chinese version of the Mini Mental Status Examination (MMSE) of 24 or higher^49^; and 5. absence of a history of taking cholinesterase inhibitors medicines.

The participants were excluded for any of the following criteria: 1. transient ischemic attack; 2. cerebral or subarachnoid hemorrhage; 3. imaging examination confirmation of a brain tumor; 4. cerebral embolism caused by atrial fibrillation with any heart diseases; 5. serious bone and joint, liver, kidney, hematopoietic system and endocrine system disease; or 6. mental disorders or dementia. At baseline, there were nine patients (three in the drug group and six in the placebo group) excluded in the fMRI data acquisition because of 1. history of pacemaker surgery, coronary intervention surgery or coronary artery bypass surgery; or 2. presence of implants. Moreover, there were three (all in the drug group) and twelve (six in the drug group and six in the placebo group) patients excluded again because of unacceptable head movement during fMRI scanning at the second and third visit, respectively.

### Neuropsychological Testing

Except for the general mental status and episodic memory, all participants were subjected to a battery of neuropsychological tests that assessed some other cognitive domains, such as working memory, attention, spatial processing, executive function and language abilities. As mentioned previously, their general mental status was assessed with the MMSE^49^. The comprehensive neuropsychological battery comprised the following 6 cognition domains (the tests used to assess each domain are in parentheses): 1, episodic memory (the AVLT^48^ and the Rey-Osterrieth Complex Figure test (ROCF) (recall)^50^); 2, working memory (the Digit Span test, which was a sub-test of the Wechsler Adults Intelligence Scale–Chinese revision); 3, processing speed (the Trail Making Test (TMT) A^51^, the Symbol Digit Modalities Test (SDMT)^52^ and the Stroop Color and Word Test (SCWT) A and B^53^); 4, visuo-spatial ability (ROCF-copy^50^ and the Clock-Drawing Test (CDT)^54^); 5, language (the Category Verbal Fluency Test (CVFT) and the Boston Naming Test (BNT)^55^); and 6, executive function (the TMT-B^51^ and the SCWT-C). We used MMSE and episodic memory as our primary outcomes due to the reported main effects of the Bushen capsules’ components on improving memory^9^,^10^,^11^,^12^,^13^ and used working memory, language and executive function as our secondary outcomes.

### Data Acquisition

MRI data were acquired using a SIEMENS TRIO 3T scanner in the Imaging Center for Brain Research, Beijing Normal University. Participants were in a supine position with their head snugly fixed by straps and foam pads to minimize head movement. Resting state data were collected using a gradient echo EPI sequence [TE = 30 ms, TR = 2000 ms, flip angle = 90°, 33 slices, slice thickness = 4 mm, in-plane matrix = 64 × 64, field of view = 256 × 256 mm^2^]. During the single-run resting acquisition, the subjects were instructed to remain awake, relax with their eyes closed, and remain as motionless as possible. The resting acquisition lasted for 8 minutes, and 240 image volumes were obtained.

### Data processing and analysis

#### Preprocessing

For each participant, the first 10 volumes were discarded to allow the participants to adapt to the magnetic field. Functional data were preprocessed using SPM and DPARSF (http://rfmri.org/DPARSF), and the processing included slice timing, within-subject interscan realignment to correct for possible movement, spatial normalization to a standard brain template in the Montreal Neurological Institute coordinate space, resampling to 3 × 3 × 3 mm^3^, and smoothing with an 8 mm full-width half-maximum Gaussian kernel.

#### Independent Component Analysis (ICA)

We performed the ICA using the group ICA toolbox (GIFT version 2.0e; http://mialab.mrn.org/software/gift/). Thirty-five components were estimated for each subject. There are three main stages to group ICA for each group of each time point analysis: (i) principal component analysis was performed for each subject for data reduction, (ii) application of the ICA algorithm, and (iii) back-reconstruction for each individual subject. After back-reconstruction, the mean spatial maps of each group at every time point were converted to z-scores for display. We focus on DMN in the current study. Then the best-fit components for the DMN were identified by visual inspection. For DMN component maps, a full factorial analysis of covariance (ANCOVA) (2 × 3) was conducted with time as the within-subject factor and group as the between-subject factor in SPM 8 (age, gender and years of education were included as covariates) (AlphaSim-corrected, *p* < 0.001).

Additionally, further analysis was performed on any significant clusters resulting from the voxel-wise comparisons. For each significant cluster, the connectivity values were extracted by averaging the intensities over all voxels within the cluster from every participant’s component map.

#### Calculation of goodness-of-fit (GOF) index

First, the GOF was calculated in MATLAB as the mean z score of all voxels within the DMN mask minus the mean z score of all voxels outside the mask (among in-brain voxels), as described previously^37^. We used the group DMN map of all subjects’ default mode component at baseline as the DMN template (FWE-corrected, P < 0.05). Positive values in this template defined the DMN mask which was used to calculate GOF index for each subject in each time point.

### Statistical Analysis

For the two treatment and placebo arms, a two-sample t-test was used to assess the differences in baseline age and years of education, and the chi-square test was used to compare the gender ratios and apolipoprotein E (*APOE*) ε4 allele frequency between the groups. For the neuropsychological assessment and the GOF index for the DMN based on fMRI data, an analysis of covariance (2 × 3) was used to test for the interaction and main effects (age, gender and years of education were included as covariates). Pearson correlation analyses were performed to explore the relationship between the changed cognitive performances and the changed regional connectivity of the significant clusters from the voxel-wise comparisons, and the changed DMN GOF index after controlling for the influences of age, gender and years of education in the drug and placebo groups separately. We calculated changed values, including cognitive performances, functional connectivity, and GOF index, for 12 months using the second visit’s values minus the baseline’s values, and for 24 months using the third visit’s values minus the baseline’s values. All statistical analyses were performed using SPSS version 22.0 for Windows.

## Additional Information

**How to cite this article**: Zhang, J. *et al*. A Two-Year Treatment of Amnestic Mild Cognitive Impairment using a Compound Chinese Medicine: A Placebo Controlled Randomized Trail. *Sci. Rep.* **6**, 28982; doi: 10.1038/srep28982 (2016).

## Supplementary Material

Supplementary Information

## Figures and Tables

**Table 1:** Demographic data for both drug and placebo groups at baseline.

	Drug group (n = 30)	Placebo group (n = 30)	F (X^2^) value	*P* value
	Mean ± SD	Mean ± SD		
Age (years)	66.00 ± 6.86	63.33 ± 6.65	1.529	0.132
Gender (male/female)^a^	16/14	12/18	1.071	0.301
*APOE* genotype (ε4 carriers/non-carriers)^a^	8/22	4/26	1.667	0.197
Education (years)	10.37 ± 3.41	10.33 ± 3.53	0.037	0.970

Abbreviation: *APOE* = apoliprotein E.

^a^The *P* values for gender and *APOE* genotype were obtained using Chi-square tests. *P* < 0.05 was considered as significant.

**Table 2:** Neuropsychological testing at three time points for both drug and placebo groups.

	Drug group (n = 30)			Placebo group (n = 30)			Group		Time		Interaction	
	Mean ± SD			Mean ± SD			Effect		Effect		Effect	
	Baseline	12 Months	24 Months	Baseline	12 Months	24 Months	F value	*P* value	F value	*P* value	F value	*P* value
General cognitive function												
MMSE	26.00 ± 2.05	27.07 ± 1.95	26.68 ± 3.58	26.67 ± 1.45	25.57 ± 2.27	25.35 ± 3.01	4.171	0.046*	1.677	0.192	11.943	<0.001*
Episodic memory												
RAVLT N5	2.47 ± 2.05	4.52 ± 2.06	4.61 ± 1.89	4.23 ± 2.51	4.30 ± 2.76	4.55 ± 2.71	0.118	0.733	3.938	0.053	11.776	0.001*
RAVLT N1N5	18.93 ± 7.02	26.07 ± 6.83	23.82 ± 5.93	25.87 ± 8.74	25.77 ± 10.60	23.82 ± 9.53	0.281	0.599	2.825	0.064	11.154	0.001*
ROCF delay recall	11.73 ± 5.84	13.17 ± 6.24	12.21 ± 5.83	9.77 ± 5.67	9.93 ± 6.01	7.66 ± 5.29	4.000	0.051	1.622	0.203	3.069	0.051
Working memory												
Digit span	10.57 ± 2.25	11.43 ± 1.78	10.21 ± 1.47	11.07 ± 1.84	10.53 ± 1.83	10.39 ± 1.61	0.053	0.818	0.596	0.444	5.937	0.018*
Backward digit span	3.97 ± 1.33	4.83 ± 0.99	4.32 ± 0.90	4.13 ± 1.17	3.77 ± 0.82	3.43 ± 0.50	5.359	0.025*	0.166	0.846	18.492	<0.001*
Language function												
BNT	23.13 ± 3.55	23.33 ± 3.44	23.50 ± 4.30	21.77 ± 3.74	22.80 ± 3.36	22.86 ± 4.91	0.756	0.388	2.393	0.128	0.532	0.469
CVFT	38.57 ± 8.05	42.43 ± 8.74	41.32 ± 8.82	42.37 ± 9.10	42.43 ± 7.68	41.93 ± 8.42	0.846	0.362	3.287	0.041*	4.699	0.011*
Visuo-spatial ability												
ROCF-copy	31.43 ± 4.59	31.61 ± 4.94	31.36 ± 5.72	30.90 ± 6.72	30.76 ± 6.32	28.76 ± 7.43	0.713	0.402	1.556	0.218	1.423	0.238
CDT	23.36 ± 3.52	23.54 ± 2.56	23.93 ± 3.98	23.48 ± 4.36	23.24 ± 4.53	23.14 ± 12.26	0.378	0.541	1.245	0.270	0.451	0.505
Processing speed												
SDMT	26.87 ± 10.30	28.77 ± 9.63	27.39 ± 10.30	31.47 ± 9.22	30.90 ± 10.60	30.00 ± 11.43	0.258	0.613	1.206	0.304	1.862	0.160
TMT part A, sec	70.23 ± 26.17	67.97 ± 24.19	74.93 ± 31.93	62.73 ± 25.04	61.20 ± 28.89	68.62 ± 30.11	0.045	0.833	5.966	0.004*	0.230	0.795
Stroop part A, sec	32.17 ± 10.00	31.73 ± 7.52	32.36 ± 8.45	30.25 ± 8.36	30.39 ± 8.92	31.00 ± 11.31	0.024	0.877	0.333	0.717	0.077	0.926
Stroop part B, sec	46.10 ± 11.49	41.00 ± 7.66	43.36 ± 8.98	41.55 ± 11.20	41.38 ± 12.54	41.21 ± 13.36	0.004	0.949	1.004	0.321	2.783	0.101
Executive function												
TMT part B, sec	220.43 ± 76.79	195.97 ± 66.73	212.82 ± 54.85	212.50 ± 63.08	196.90 ± 89.20	212.69 ± 97.56	0.221	0.640	1.123	0.329	0.186	0.830
Stroop part C, sec	93.33 ± 23.71	80.80 ± 17.67	84.79 ± 18.36	81.14 ± 27.93	81.72 ± 28.96	83.50 ± 31.23	0.009	0.924	1.762	0.177	3.128	0.048*
TMT part B-A, sec	150.20 ± 66.69	128.00 ± 57.15	137.89 ± 46.26	149.77 ± 50.97	135.70 ± 65.41	144.07 ± 79.05	0.522	0.473	1.697	0.198	0.129	0.721
Stroop part C-B, sec	47.23 ± 20.03	39.80 ± 15.81	41.43 ± 18.24	39.59 ± 21.58	40.35 ± 20.44	42.29 ± 25.06	0.007	0.932	1.285	0.281	1.693	0.189

Abbreviation: MMSE = Mini-Mental Status Examination; AVLT = Auditory Verbal Learning Test; ROCF = Rey-Osterrieth Complex Figure test; CDT = Clock-Drawing Test; CVFT = Category Verbal Fluency Test; BNT = Boston Naming Test; SDMT = Symbol Digit Modalities Test; SCWT = Stroop Color and Word Test; TMT = Trail Making Test. The comparisons of the neuropsychological scores between the two groups (drug and placebo) within three time points (baseline, 12 months and 24 months) were performed with the mixed-model ANOVA (2 × 3). Thereafter, the multiple comparisons were performed by F tests. *P* < 0.05 was considered as significant.

## References

[b1] Petersen R. C. . Current concepts in mild cognitive impairment. Arch Neurol 58, 1985–1992, doi: 10.1001/archneur.58.12.1985 (2001). 11735772

[b2] Butchart J. . Etanercept in Alzheimer disease: A randomized, placebo-controlled, double-blind, phase 2 trial. Neurology 84, 2161–2168, doi: 10.1212/WNL.0000000000001617 (2015). 25934853 PMC4451045

[b3] Doody R. S. . Phase 3 trials of solanezumab for mild-to-moderate Alzheimer’s disease. N Engl J Med 370, 311–321, doi: 10.1056/NEJMoa1312889 (2014). 24450890

[b4] Likitjaroen Y. . Longitudinal changes of fractional anisotropy in Alzheimer’s disease patients treated with galantamine: a 12-month randomized, placebo-controlled, double-blinded study. Eur Arch Psychiatry Clin Neurosci 262, 341–350, doi: 10.1007/s00406-011-0234-2 (2012). 21818628

[b5] Salloway S. . Two phase 3 trials of bapineuzumab in mild-to-moderate Alzheimer’s disease. N Engl J Med 370, 322–333, doi: 10.1056/NEJMoa1304839 (2014). 24450891 PMC4159618

[b6] Schmidt R. . Longitudinal multimodal imaging in mild to moderate Alzheimer disease: a pilot study with memantine. J Neurol Neurosurg Psychiatry 79, 1312–1317, doi: 10.1136/jnnp.2007.141648 (2008). 18586865 PMC2582338

[b7] Wu T. Y., Chen C. P. & Jinn T. R. Traditional Chinese medicines and Alzheimer’s disease. Taiwanese journal of obstetrics & gynecology 50, 131–135, doi: 10.1016/j.tjog.2011.04.004 (2011). 21791295

[b8] Backman L., Small B. J. & Fratiglioni L. Stability of the preclinical episodic memory deficit in Alzheimer’s disease. Brain 124, 96–102 (2001). 11133790 10.1093/brain/124.1.96

[b9] Choi J. G. . Cistanches Herba enhances learning and memory by inducing nerve growth factor. Behav Brain Res 216, 652–658, doi: 10.1016/j.bbr.2010.09.008 (2011). 20849880

[b10] Ahn S. M., Kim Y. R., Kim H. N., Shin H. K. & Choi B. T. Beneficial Effects of Polygonum multiflorum on Hippocampal Neuronal Cells and Mouse Focal Cerebral Ischemia. Am J Chin Med 43, 637–651 (2015). 26119951 10.1142/S0192415X15500391

[b11] Heo H. . Memory improvement in ibotenic acid induced model rats by extracts of Scutellaria baicalensis. J Ethnopharmacol 122, 20–27, doi: 10.1016/j.jep.2008.11.026 (2009). 19111602

[b12] Kou J., Zhu D. & Yan Y. Neuroprotective effects of the aqueous extract of the Chinese medicine Danggui-Shaoyao-san on aged mice. J Ethnopharmacol 97, 313–318 (2005). 15707771 10.1016/j.jep.2004.11.020

[b13] Zhao H. . Long-term ginsenoside consumption prevents memory loss in aged SAMP8 mice by decreasing oxidative stress and up-regulating the plasticity-related proteins in hippocampus. Brain Res 1256, 111–122, doi: 10.1016/j.brainres.2008.12.031 (2009). 19133247

[b14] Zhang J. . The Effects of Bushen Capsule on Episodic Memory in Amnestic Mild Cognitive Impairment Patients: A Pilot Placebo Controlled fMRI Study. J Alzheimers Dis 46, 665–676, doi: 10.3233/JAD-150004 (2015). 25854932

[b15] Zhang J. . The effects of CCRC on cognition and brain activity in aMCI patients: a pilot placebo controlled BOLD fMRI study. Curr Alzheimer Res 11, 484–493, doi: 10.2174/1567205011666140505095939 (2014). 24801219

[b16] Weiner M. W. . Magnetic resonance imaging and neuropsychological results from a trial of memantine in Alzheimer’s disease. Alzheimers Dement 7, 425–435 (2011). 21646051 10.1016/j.jalz.2010.09.003

[b17] Damoiseaux J. S. . Consistent resting-state networks across healthy subjects. Proc Natl Acad Sci USA 103, 13848–13853, doi: 10.1073/pnas.0601417103 (2006). 16945915 PMC1564249

[b18] Dai Z. . Identifying and Mapping Connectivity Patterns of Brain Network Hubs in Alzheimer’s Disease. Cereb Cortex, doi: 10.1093/cercor/bhu246 (2014). 25331602

[b19] Toussaint P. J. . Characteristics of the default mode functional connectivity in normal ageing and Alzheimer’s disease using resting state fMRI with a combined approach of entropy-based and graph theoretical measurements. Neuroimage 101, 778–786 (2014). 25111470 10.1016/j.neuroimage.2014.08.003

[b20] Wang Y. . Altered default mode network connectivity in older adults with cognitive complaints and amnestic mild cognitive impairment. J Alzheimers Dis 35, 751–760, doi: 10.3233/JAD-130080 (2013). 23481685 PMC3962306

[b21] Fratiglioni L. . Incidence of dementia and major subtypes in Europe: A collaborative study of population-based cohorts. Neurologic Diseases in the Elderly Research Group. Neurology 54, S10–15 (2000). 10854355

[b22] Cockrell J. R. & Folstein M. F. Mini-mental state examination. Principles and practice of geriatric psychiatry 140–141 (2002).

[b23] Perneczky R. The appropriateness of short cognitive tests for the identification of mild cognitive impairment and mild dementia. Aktuelle Neurologie 30, 114–117 (2003).

[b24] Courtney C. . Long-term donepezil treatment in 565 patients with Alzheimer’s disease (AD2000): randomised double-blind trial. Lancet 363, 2105–2115 (2004). 15220031 10.1016/S0140-6736(04)16499-4

[b25] Andersson C. . Identifying patients at high and low risk of cognitive decline using Rey Auditory Verbal Learning Test among middle-aged memory clinic outpatients. Dement Geriatr Cogn Disord 21, 251–259 (2006). 16465053 10.1159/000091398

[b26] Viola S. . New brain reperfusion rehabilitation therapy improves cognitive impairment in mild Alzheimer’s disease: a prospective, controlled, open-label 12-month study with NIRS correlates. Aging Clin Exp Res 26, 417–425 (2014). 24338518 10.1007/s40520-013-0185-8

[b27] Hogervorst E., Yaffe K., Richards M. & Huppert F. A. Hormone replacement therapy to maintain cognitive function in women with dementia. Cochrane Database Syst Rev 21, doi: 10.1002/14651858.CD003799.pub2 (2009). 12137718

[b28] Baker L. D. . Effects of growth hormone-releasing hormone on cognitive function in adults with mild cognitive impairment and healthy older adults: results of a controlled trial. Arch Neurol 69, 1420–1429 (2012). 22869065 10.1001/archneurol.2012.1970 PMC3764914

[b29] Tobinick E. L. & Gross H. Rapid improvement in verbal fluency and aphasia following perispinal etanercept in Alzheimer’s disease. BMC Neurol 8, 1471–2377 (2008). 10.1186/1471-2377-8-27 PMC2500042 18644112

[b30] Cho K. . A preliminary study on the inhibitory effect of Chunghyul-dan on stroke recurrence in patients with small vessel disease. Neurol Res 30, 655–658 (2008). 18498682 10.1179/174313208X305382

[b31] May B. H. . Herbal medicine for dementia: a systematic review. Phytother Res 23, 447–459, doi: 10.1002/ptr.2656 (2009). 19086008

[b32] Wei D. . The therapeutic effect of Xueshuan Xinmai tablets on memory injury and brain activity in post-stroke patients: a pilot placebo controlled fMRI study. Int J Clin Exp Med 8, 7507–7516 (2015). 26221294 PMC4509239

[b33] Yu L. . Chinese herbal medicine for patients with mild to moderate Alzheimer disease based on syndrome differentiation: a randomized controlled trial. Zhong Xi Yi Jie He Xue Bao 10, 766–776 (2012). 22805083 10.3736/jcim20120707

[b34] Raichle M. E. . A default mode of brain function. Proc Natl Acad Sci USA 98, 676–682, doi: 10.1073/pnas.98.2.676 (2001). 11209064 PMC14647

[b35] Qi Z. . Impairment and compensation coexist in amnestic MCI default mode network. Neuroimage 50, 48–55, doi: 10.1016/j.neuroimage.2009.12.025 (2010). 20006713

[b36] Bai F. . Specifically progressive deficits of brain functional marker in amnestic type mild cognitive impairment. PLoS One 6, e24271, doi: 10.1371/journal.pone.0024271 (2011). 21935394 PMC3174167

[b37] Greicius M. D., Srivastava G., Reiss A. L. & Menon V. Default-mode network activity distinguishes Alzheimer’s disease from healthy aging: evidence from functional MRI. Proc Natl Acad Sci USA 101, 4637–4642, doi: 10.1073/pnas.0308627101 (2004). 15070770 PMC384799

[b38] Petrella J. R., Sheldon F. C., Prince S. E., Calhoun V. D. & Doraiswamy P. M. Default mode network connectivity in stable vs progressive mild cognitive impairment. Neurology 76, 511–517, doi: 10.1212/WNL.0b013e31820af94e (2011). 21228297 PMC3053179

[b39] Cha J. . Functional alteration patterns of default mode networks: comparisons of normal aging, amnestic mild cognitive impairment and Alzheimer’s disease. Eur J Neurosci 37, 1916–1924 (2013). 23773060 10.1111/ejn.12177 PMC3694739

[b40] Daamen M. . Working memory in preterm-born adults: load-dependent compensatory activity of the posterior default mode network. Hum Brain Mapp 36, 1121–1137 (2015). 25413496 10.1002/hbm.22691 PMC6869743

[b41] Bai Y. N. . Effect of jianpi tongluo jiedu recipe on expression levels of COX-2, NF-kappaBp65, and Bcl-2 in gastric mucosa of patients with precancerous lesions of gastric cancer. Zhongguo Zhong Xi Yi Jie He Za Zhi 35, 167–173 (2015). 25881460

[b42] Wu L. F., Xing Y., Guan Y. L., Liu Z. Q. & Zhang W. S. Protective effect of jiedu tongluo injection on cerebral edema in rats with lesion of cerebral ischemia/reperfusion. Zhongguo Zhong Yao Za Zhi 39, 1088–1092 (2014). 24956856

[b43] Xuan G. & Liu C. [Research on the effect of phenylethanoid glycosides (PEG) of the Cistanche deserticola on anti-aging in aged mice induced by D-galactose]. Zhong yao cai= Zhongyaocai= Journal of Chinese medicinal materials 31, 1385–1388 (2008). 19180965

[b44] Su R., Han Z.-y. & Fan J.-p. Effect of Fufang Congrong Yizhi Capsule on myristoylated alanine-rich C-kinase substrate (MARCKS) mRNA level in hippocampus of old dementia rats [J]. China Journal of Traditional Chinese Medicine and Pharmacy 4, 046 (2010).

[b45] Cheung T. S. . Therapeutic Effects of Herbal Chemicals in Traditional Chinese Medicine on Alzheimer’s Disease. Curr Med Chem 22, 2392–2403 (2015). 25989911 10.2174/0929867322666150520095509

[b46] Zhang Y. . Cognitive Improvement during Treatment for Mild Alzheimer’s Disease with a Chinese Herbal Formula: A Randomized Controlled Trial. PLoS One 10, e0130353, doi: 10.1371/journal.pone.0130353 (2015). 26076022 PMC4468068

[b47] Li X. . Prevalence of and potential risk factors for mild cognitive impairment in community-dwelling residents of Beijing. J Am Geriatr Soc 61, 2111–2119 (2013). 24479143 10.1111/jgs.12552

[b48] Rosenberg S. J., Ryan J. J. & Prifitera A. Rey auditory‐verbal learning test performance of patients with and without memory impairment. Journal of clinical psychology 40, 785–787 (1984). 6746989 10.1002/1097-4679(198405)40:3<785::aid-jclp2270400325>3.0.co;2-4

[b49] Zhang M. . The prevalence of dementia and Alzheimer’s disease in Shanghai, China: impact of age, gender, and education. Annals of neurology 27, 428–437 (1990). 2353798 10.1002/ana.410270412

[b50] Rey A. L-examen psychologique dans les cas d’encephalopathie traumatique. Arch Psychologie 1941, 286–340 (1941).

[b51] Reitan R. Validity of the trail making test as an indicator of organic brain damage. Percept Mot Skills 8, 271–276 (1958).

[b52] Sheridan L. K. . Normative Symbol Digit Modalities Test performance in a community-based sample. Arch Clin Neuropsychol 21, 23–28, doi: 10.1016/j.acn.2005.07.003 (2006). 16139470

[b53] Guo Q. . Application of Stroop color-word test on Chinese elderly patients with mild cognitive impairment and mild Alzheimer’s dementia. Chinese Journal of Neuromedicine 4, 701–704 (2005).

[b54] Rouleau I., Salmon D. P., Butters N., Kennedy C. & McGuire K. Quantitative and qualitative analyses of clock drawings in Alzheimer’s and Huntington’s disease. Brain Cogn 18, 70–87 (1992). 1543577 10.1016/0278-2626(92)90112-y

[b55] Guo Q. Boston Naming Test in Chinese elderly, patient with mild cognitive impairment and Alzheimer’s dementia. Chinese Mental Health Journal 20, 81 (2006).

