# Corrigendum: A Two-Year Treatment of Amnestic Mild Cognitive Impairment using a Compound Chinese Medicine: A Placebo Controlled Randomized Trial

**DOI:** 10.1038/srep30511

**Published:** 2016-08-19

**Authors:** Junying Zhang, Zhen Liu, Huamin Zhang, Caishui Yang, He Li, Xin Li, Kewei Chen, Zhanjun Zhang

Scientific Reports 6: Article number: 2898210.1038/srep28982; published online: 07 04 2016; updated: 08 19 2016.

The original version of this Article contained an error in the title of the paper, where the word “Trial” was incorrectly given as “Trail”. This has now been corrected in the PDF and HTML versions of the Article.

