# Supplementary material for: A Two-Year Treatment of Amnestic Mild Cognitive Impairment using a Compound Chinese Medicine: A Placebo Controlled Randomized Trail
Source: Sci Rep. 2016 Jul 4;6:28982. doi: 10.1038/srep28982 (PMC4931444; doi:10.1038/srep28982)
Supplement: Supplementary Information [file srep28982-s1.doc]

A Two-Year Treatment of Amnestic Mild Cognitive Impairment using a Compound Chinese Medicine: A Placebo Controlled Randomized Trail

Junying Zhang, MD^1,2,5^, Zhen Liu, MS^1,2,5^, Huamin Zhang, MD^3,2,5^, Caishui Yang, MS^1,2^, He Li, PhD^3,2^, Xin Li, PhD^1,2^, Kewei Chen, PhD^4,2^ and Zhanjun Zhang, MD^1,2*^

^1^ State Key Laboratory of Cognitive Neuroscience and Learning & IDG/McGovern Institute for Brain Research, Beijing Normal University, Beijing 100875, P. R. China;

^2^ BABRI Centre, Beijing Normal University, Beijing 100875, P. R. China;

^3^ Institute of Information on Traditional Chinese Medicine, China Academy of Chinese Medical Sciences, Beijing 100700, P. R. China;

^4^ Banner Alzheimer's Institute, Phoenix, AZ 85006, USA;

^5^ These authors contributed to the work equally.

Correspondence to: Prof Zhanjun Zhang, State Key Laboratory of Cognitive Neuroscience and Learning& IDG/McGovern Institute for Brain Research, Beijing Normal University, Beijing 100875, P. R. China. Tel: 86-10-58802005, fax: 86-10-58802005, email: [zhang_rzs@bnu.edu.cn](mailto:zhang_rzs@bnu.edu.cn).


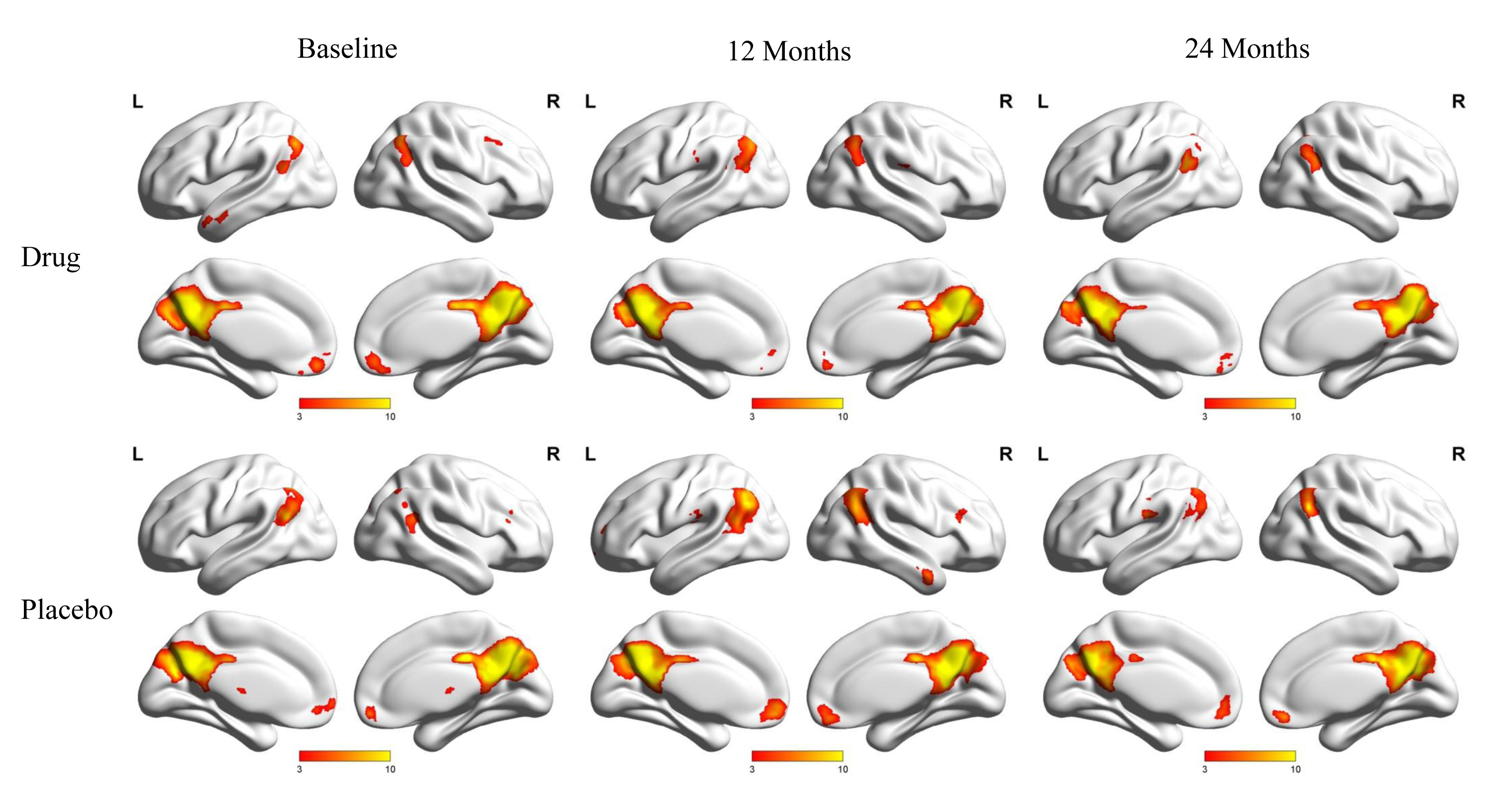


Figure e-1 Spatial maps of the DMN of the drug group and the placebo group at every visit are shown in red–yellow.

DMN, default mode network.
